# The relationship between the chemical composition and the quality of the soybean film during peeling process

**DOI:** 10.1002/fsn3.1791

**Published:** 2020-07-27

**Authors:** Yin‐Yi Ding, Juanjuan Li, Cheng Wu, Yuexi Yang, Zhenyu Gu

**Affiliations:** ^1^ School of Food Science and Biotechnology Zhejiang Gongshang University Hangzhou China; ^2^ National Experimental Teaching Demonstration Center of Food Engineering and Quality and Safety Hangzhou China; ^3^ Shanghai Institutes for Biological Sciences Chinese Academy of Sciences Zhejiang Gongshang University Joint Centre for Food and Nutrition Research Zhejiang Gongshang University Hangzhou China

**Keywords:** Maillard reaction, main component, sensory evaluation, soybean film, soybean milk

## Abstract

Soybean film is a traditional nonfermented soy product in China. It is a film formed on the surface of the soymilk during heating process. The nutrient components of soymilk will affect the quality of the soybean film. The results of this study showed that during the peeling process, the proportion of protein and carbohydrate in soymilk decreased, and the proportion of lipid increased. The mechanical properties (fracture extension and tensile strength) of the soybean film decreased during the peeling process. During the heating treatment, the Maillard reaction occurred and its intermediate products accumulated, which caused the change in soybean film color. White globular protein granules (<100 nm) existed on the surface of the soybean film. The lipid that was not wrapped by the protein network structure was exposed, and the evaporation of water led to the formation of black and gray holes on the skin (<500 nm). In addition, the results of correlation analysis showed that the changes in color, taste, and odor, as well as the mechanical properties of the skin, were all related to the changes in nutrients in the soybean film during peeling. This research provided a deeper understanding of the quality change in the soybean milk and the soybean film during the heating process.

## Graphical abstract

This manuscript describes the basic ingredients of soymilk will affect the quality of the soybean film. This paper mainly studies the changes in main nutrients and basic physicochemical properties of soymilk and soybean film after continuous peeling and forming at the same time interval. It provides theoretical basis and practical experience for producing better quality soybean film.

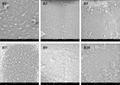

## 1 INTRODUCTION

Soybean film is a thin film formed on the surface of soybean milk during heating process. During the heating process, soybean protein denatures and forms hydrophobic bonds with lipid membrane (Kim, Weller, Hanna, & Aristippos, 2002). The main components in the soybean film are protein, lipid, and carbohydrate (Wang et al., 2018). Soybean film also contains a lot of minerals, including calcium, iron, magnesium, and zinc (Wu & Bates, 2010).

As the temperature rises, hydrophobic groups of the soy protein are exposed, and cross‐linking occurs between molecules, forming a network structure, and finally forming a soybean film structure (Kim et al., 2002). In the process of the soybean film formation, the distribution of protein and lipid has a certain regularity (Banerjee & Chen, 1995). The lipid interacts with denatured proteins or other peptide chains to form a lipid–protein complex (Shun‐Tang, Tomotada, & Mikami, 1997). The composition of the soybean milk can affect the formatting process and the quality of the film, including color, the mechanical properties, and the microstructure (Galietta, Di Gioia, Stéphane, & Bernard, 1998; Yeming & Tomotada, 2010). However, very little research has been done on the relationship between the properties of soymilk and the quality of the soybean film.

In this study, the physical and chemical properties of soymilk and soybean film during the process of continuous peeling were investigated, in order to provide a better theoretical guidance for the industrial production of soybean film.

## 2 MATERIALS AND METHODS

### 2.1 Materials

Soybeans harvested from Liaoning Province of China were obtained from a local grocery store and then stored at 4℃ after sealed up in vacuum plastic bags for further research. 8‐Anilino‐1‐naphthalene sulfonic acid was purchased from J&K Scientific Co., Ltd. (Beijing, China); All reagents were of analytical grade (purity > 99.7%).

### 2.2 Preparation of soybean films

The raw soymilk for further heating treatment was prepared by the following steps:

Step 1: Soak 100 ± 0.05 g of soybean in 400 ml of distilled water at 25°C for 12 hr.
Step 2: Drain the water from the soaked soybeans and ground with 700 ml distilled water using a soymilk grinder (Model JYL‐Y5, Joyoung Co., China) for 4 min to obtain the slurry.
Step 3: Heat the soybean milk on the induction cooker and let it boil for 3 min. The slurry was filtered with 4 layers of gauze to discard the bean dreg and reserve the soybean milk.
Step 4: Transfer 200 ml of filtered soymilk to a container and heat to 85°C. One piece of film was peeled every 20 min until a complete film could not be formed. After each peeling process, take 30 ml of soymilk for the physical and chemical indicators, named A1, A2, A3, A4, A5, A6, A7, A8, A9, and A10. Soybean film was named after the order of B1, B2, B3, B4, B5, B6, B7, B8, B9, and B10.

### 2.3 Chemical analyses

#### 2.3.1 Determination of protein content

The determination of protein content was carried out by using the Kjeldahl method according to Lee (1995).

#### 2.3.2 Determination of lipid content

The determination of lipid content was carried out according to Lee's research (Lee, 1995).

#### 2.3.3 Determination of total carbohydrate

The total carbohydrate content was determined by the 3, 5‐dinitrosalicylic acid method (Lindsay, 1973).

### 2.4 SDS‐PAGE

The method of SDS‐PAGE was referred to Laemmli (1970). The concentrations of the stacking gel and separating gel were 5% and 12%, respectively. The buffer in the reservoirs contained 0.025 M Tris, 0.192 M glycine, and 0.1% (w/v) SDS, and the buffer in the stacking and running gels was 0.125 M Tris‐HCl (pH 6.8) and 0.38 M Tris‐HCl (pH 8.8), respectively. The sample solution containing 0.25 M Tris‐HCl (pH 6.8), 1% (w/v) SDS, and 2% (w/v) DTT was mixed with the same volume of glycerol containing bromophenol blue, and boiled for 5 min. Then, 10 μl of the boiled sample was loaded into each sample well. After electrophoresis, the gels were stained with dye, which contained Coomassie Brilliant Blue R‐250 (0.1%, w/v), methanol (20%, v/v), and acetic acid (10%, v/v) in distilled water.

### 2.5 Mechanical properties

Mechanical properties are important indicators for evaluating the quality of the soybean film (Sebti, Chollet, Degraeve, Noel, & Peyrol, 2007). There are two indicators widely used to evaluate the mechanical properties of soy products and noodle products, tensile strength (TS), and elongation at break (Δ*E*; Shellhammer & Krochta, 2006).

According to Valenzuela's method (Valenzuela, Abugoch, & Tapia, 2013),the soybean film was cut into strips of 2 cm × 4 cm, and tensile test was performed using a texture tester to determine the maximum breaking tension (g), TS, and Δ*E* of the film. The membrane was rehydrated before the measurement. At least three parallel samples of each group were measured.

The tensile strength was calculated according to Equation (1):

(1) $$\text{TS}\left(g/{\text{cm}}^{2}\right)=F/S$$

where *F* was the maximum tension when the sample broke, g; *S* was the cross‐sectional area of the sample, cm^2^.

The elongation at break was calculated according to Equation (2):

(2) $$E\left(\%\right)=\frac{L_{1}‐L_{0}}{L_{0}}\times 100$$

where *L*_1_ was the length of the broken film, mm; *L*_0_ was the original length of the film, mm.

### 2.6 Determination of dry material loss rate and rehydration rate

The 2 cm × 2 cm soybean film was boiled in a water bath at 85°C for 15 min and then drained for 5 min and weighted (*m*_1_). The film was subsequently dried in an oven at 105°C for 3 hr and weighed (*m*_2_). The dry matter lost rate was calculated according to Equation (3). The rehydration rate of soybean film was calculated according to Equation (4).

(3) $$\text{Dry matter lost (\textbackslash{}\% )}=\frac{m_{0}\times \left(1‐\omega \right)‐m_{2}}{m_{0}\times \left(1‐\omega \right)}\times 100$$

(4) $$\text{Rehydration rate (\textbackslash{}\% )}=\frac{m_{1}‐m_{0}}{m_{0}}\times 100$$

where *m*_0_ was the weight of soybean film before boiling; *ω* was the moisture content of soybean film (%).

### 2.7 Determination of browning index of soybean milk

The lyophilized soybean milk powder was mixed with 30% methanol, and magnetically stirred for 30 min in a 40°C water bath. After centrifugation at 6577 *g* for 10 min, the absorbance of the supernatant was measured at the wavelength of 420 nm with an ultraviolet–visible spectrophotometer to indicate the browning index.

### 2.8 Determination of color of the soybean film

The soybean film that had been peeled was wiped off with kitchen paper to make it spread flat, and the color was measured using an automatic color difference meter (CR‐400, Ke Sheng Xing Instrument Co. LTD, China). The L* value (brightness) was read at five different positions for each sample. The a* value (red/green) and the b* value (yellow/blue) were used as color indicators.

### 2.9 Determination of intermediate products in the Maillard reactions

The Maillard reaction could be divided into three major periods: initial period, intermediate period, and final period (Topova, Bojilov, Dagnon, & Argirov, 2014). Among them, carbonyl and dicarbonyl compounds, as well as some intermediate substances, such as furfural and ketone, were generated by Amadori reconfiguration and Strecker degradation reaction (Varoujan, 2003). These intermediates had absorption peaks under the ultraviolet–visible wavelength of 294 nm (Ajandouz, Desseaux, Tazi, & Puigserver, 2008). The content of 5‐hydroxymethylfurfural (5‐HMF) and furfural was determined by high‐performance liquid chromatography at wavelength of 280 nm (Li, Xu, Zhang, & Wang, 2017).

### 2.10 Scanning electron microscopy

According to Noh's (Noh, Park, Pak, Hong, & Yun, 2005) method, the soybean film was cut into a size of 0.5 cm × 0.5 cm, and fixed on a sample period with a special double‐sided tape, and then sprayed with gold. The surface morphology of the sample was observed by scanning electron microscopy. The magnification of the electron microscope was 10,000×.

### 2.11 Sensory evaluation of soybean film

Twenty food sensory professionals were selected for the experiment. The soybean film for sensory evaluation was prepared for rehydration 5 min before the experiment and cut into a square of 4 cm × 4 cm. In the sensory evaluation, one set of samples was completed before the next set was given. Sensory indicators were judged according to Table 1.

**TABLE 1:** Standard of sensory evaluation of soybean film

Sensory index	Evaluation	Score
Color	Bright yellow color with natural grease sheen	7–9
	Dark color and less shiny	4–6
	The color appears gray yellow, deep yellow, or brown yellow, dark, and lusterless	1–3
Odor	Has the fresh and full‐bodied soybean flavor, does not have the bad smell	7–9
	There is a certain natural soybean flavor, a little bad smell	4–6
	No soybean flavor, heavy bad odor (peculiar smell)	1–3
Taste	Good toughness, excellent taste	7–9
	General toughness, certain tensile properties, general taste	4–6
	Poor toughness, easy to break, poor taste	1–3
Acceptability	Perfect, very acceptable	7–9
	Slightly defective, acceptable	4–6
	Unacceptable	1–3

### 2.12 Statistical analysis

All data were the average of three independent trials. The statistical analysis was performed with SPSS 22.0. The correlation analysis and cluster analysis were performed with SPSS 22.0. The results were reported as mean ± standard deviations. ANOVA was conducted, and differences between individual value were deemed to be significant at *p* < .05.

## 3 RESULTS AND DISCUSSION

### 3.1 Basic composition of the soybean films and soymilk

Tables 2 and 3 show the changes in protein, total carbohydrate, and lipid content in the soymilk and soybean film during the continuous peeling process. The raw milk had not been filtered, and it contained impurities such as bean dregs, resulting in lower protein content than other soymilk that had been filtered and boiled. From the beginning of the heating process, protein content in the soybean milk decreased from 43.63% to 42.13%, and the protein content in the soybean film was also gradually reduced with the increase in the number of peeling. During the formation of soybean film, the water continuously evaporated and dissipated at high temperature, maintaining the balance of the soymilk system. As the protein continued to form soybean film, the degree of protein denaturation increases at high temperatures, leading to protein aggregation (Siran, Nayeon, Wallace, & Yookyung, 2018).

**TABLE 2:** Changes in the basic composition of soybean milk during the film peeling

	Protein (%)	Total carbohydrate (%)	Lipid (%)
A0	43.63 ± 0.03^a^	0.79 ± 0.01^f^	17.23 ± 0.21^e^
A1	43.38 ± 0.1^b^	0.83 ± 0.01^e^	17.74 ± 0.15^e^
A2	43.11 ± 0.27^c^	0.87 ± 0.02^d^	19.38 ± 0.15^d^
A3	43.29 ± 0.08^bc^	0.88 ± 0.01^c^	21.56 ± 0.49^cd^
A4	43.33 ± 0.16^bc^	0.73 ± 0.01^g^	23.11 ± 0.61^c^
A5	42.71 ± 0.17^d^	0.71 ± 0.01^g^	23.19 ± 1.42^bcde^
A6	42.74 ± 0.06^d^	0.85 ± 0.01d^e^	26.04 ± 1.28^bcd^
A7	42.35 ± 0.16^ef^	0.85 ± 0.01^de^	27.04 ± 0.61^b^
A8	42.41 ± 0.13^e^	0.96 ± 0.01^a^	28.62 ± 0.02^b^
A9	42.42 ± 0.14^e^	0.95 ± 0.01^a^	29.28 ± 0.24^ab^
A10	42.13 ± 0.07^f^	0.92 ± 0.02^b^	29.44 ± 0.11^a^

Note

Different letters indicate significant differences (*p* < .05)

**TABLE 3:** Changes in the basic composition of soybean film during the film peeling

	Protein (%)	Total carbohydrate (%)	Lipid (%)
B1	47.27 ± 0.15^c^	1.04 ± 0.14^b^	26.85 ± 0.01^a^
B2	48.09 ± 0.12^a^	1.05 ± 0.35^b^	26.71 ± 0.05^a^
B3	47.94 ± 0.03^ab^	1.73 ± 0.12^a^	25.91 ± 0.57^abc^
B4	47.83 ± 0.14^ab^	3.39 ± 0.09^a^	25.73 ± 0.07^b^
B5	47.81 ± 0.1^ab^	3.05 ± 0.79^a^	25.31 ± 0.29^bc^
B6	47.65 ± 0.32^b^	4.16 ± 0.23^a^	24.31 ± 0.23^c^
B7	47.59 ± 0.43^bc^	4.65 ± 0.57^a^	22.51 ± 0.11^de^
B8	47.11 ± 0.27^cd^	3.84 ± 0.11^a^	21.85 ± 0.26^e^
B9	46.83 ± 0.05^d^	3.69 ± 0.31^a^	21.21 ± 0.25^e^
B10	46.35 ± 0.06^e^	3.18 ± 0.01^ab^	20.39 ± 0.06^e^

Note

Different letters indicate significant differences (*p* < .05)

Lipid is one of the main components in the formation of the soybean film, which also has an important impact on the quality of soybean film (Lindsay, 1973). In this study, Tables 2 and 3 show the changes in lipid content in soymilk and soybean film during peeling. The lipid content in soymilk increased and tended to be flat in the later period. It showed that the amount of lipid needed to form the soybean film was lower than protein required. The formation of soybean film resulted in the decrease in lipid content in soymilk. In addition, as the peeling process continued, the proportion of lipid in the composition of the film decreased. Combined with the change in protein content, it showed that in the process of continuous film formation, the protein was gradually denatured under continuous heat preservation. During the peeling process, the ability of soybean milk to form a network structure decreased, and the strength of network structure also decreased, which also led to the continuous reduction of lipid wrapped in the network structure.

The total carbohydrate content in the soymilk was significantly lower than that of the unheated soybean milk, and the total carbohydrate content in the soymilk decreased as the heating progressed, and reached the lowest level in the fifth film. Since the slurry was not filtered, the carbohydrate content was significantly higher than the filtered soybean milk. Since the formation of the soybean film and the heating process kept at a relatively high temperature, and the reducing sugar might react with the amino group of the soybean film at the high temperature, the carbohydrate in the soybean milk gradually decreased. In the late period of heat preservation, the carbohydrate content in the soybean milk was maintained at a relatively low level, which might be due to the film‐forming efficiency that had been greatly reduced in the later period.

In addition, the change in total carbohydrate in the soybean film showed a trend of increasing first and then decreasing, and reached the maximum level at the sixth sheet. As the peeling process continued, the composition of the soymilk changed, and the content of the initial substance was decreased, resulting in a decrease in film formation efficiency and a reduction in the amount of saccharide entering the soybean film (Hernandez‐Izquierdo & Krochta, 2010).

### 3.2 The effect of soluble protein on soybean film formation

The soy protein composition in soymilk can be divided into four components: 2S, 7S, 11S, and 15S according to the mode of centrifugal sedimentation. Among them, 2S and 15S accounted for a small proportion, while 7S (β‐conglycinin) and 11S (glycinin) were dominant, accounting for about 80% of soybean protein (Duanquan, Kelly, & L., Maidannyka, V., Miao, S., 2020). 7S was a protein in the form of a trimer structure composed of three subunits, including α', α, and β. 11S is composed of 6 acidic subunits A and 6 basic subunits B, and the A and B subunits were linked together by disulfide bonds (Hongkang, Lite, Eizo, & Seiichiro, 2005).

**FIGURE 1:**
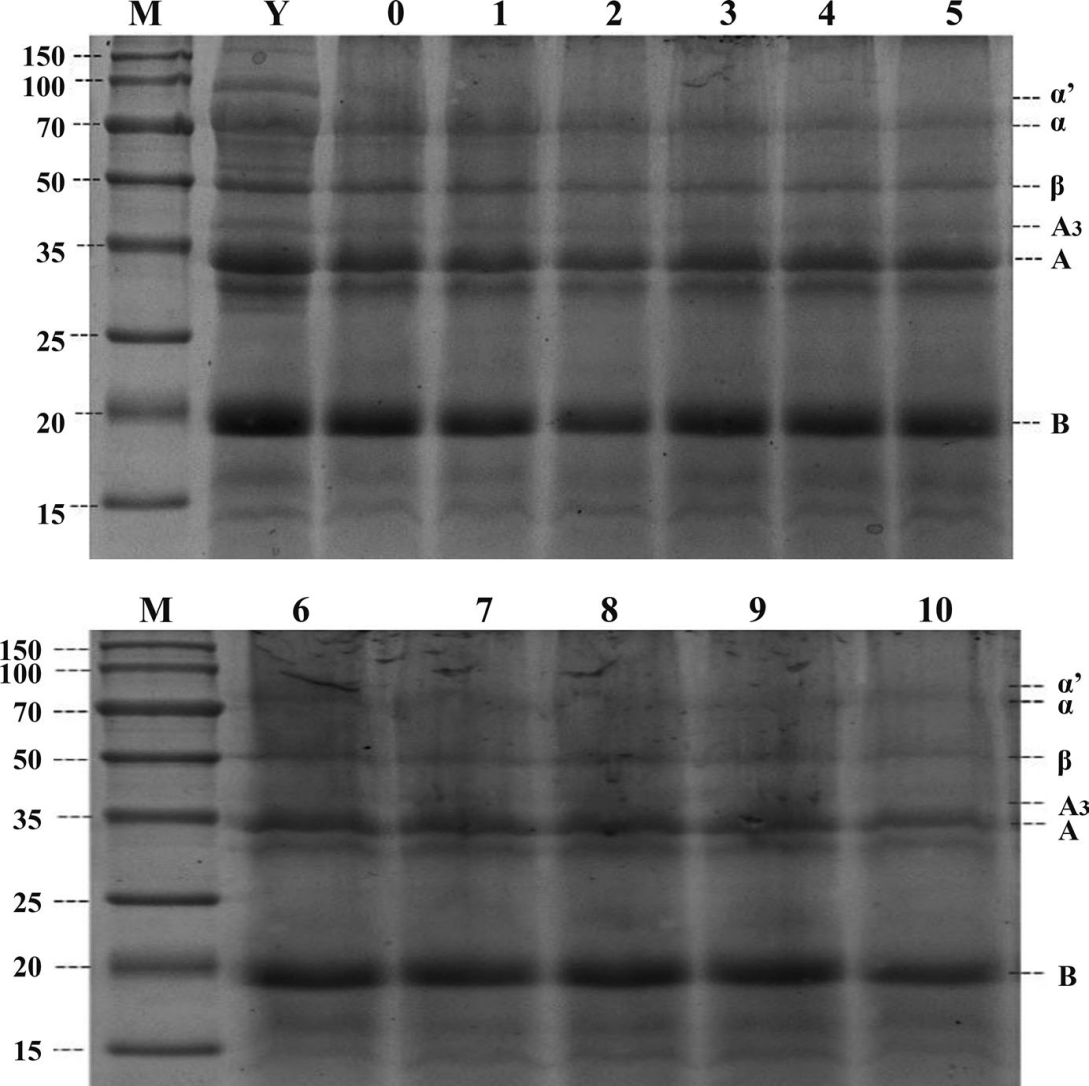
SDS‐PAGE profile of soymilk during the film picking. M: maker; Y: raw soymilk; 1–10: soymilk during the film picking; α, α’, and β: subunits of β‐conglycinin; A, A3, and B: acidic and basic subunits of glycinin

Figure 1 shows that the 7S component in the soymilk gradually decreases during the peeling process, while the acidic subunit A in the 11S subunit was obvious. The basic subunit B was slightly deeper but not obvious. The 7S subunit strips were lighter than the heated stock. In the process of peeling off, the ratio of 11S/7S increased, and the bands of α’, α, and β were lightened.

Some researchers separated a single subunit into a separate film, and found that only the group of α’ and α subunits could form a film. The α’ and α subunits had amphiphilic structure, so α’/α had significant effect on the film formation (Xiuzhi et al., 2007). In the process of peeling, as the holding time increased, the soybean film was fully heat‐denatured, and the α’ and α subunits were concentrated by heating to some extent, and participate in the film‐forming process, resulting in a decrease in the content in the soybean milk. In addition, the α’ and α subunits would gradually reach the critical micelle concentration during the heating process (Scholz, Behnke, & Resch‐Genger, 2018), resulting in the formation of larger micelles, which would promote the formation of protein network structure (JiSu, Mi‐Ja, & JaeHwan, 2018).

### 3.3 Changes in color of soybean film and soymilk during peeling

**FIGURE 2:**
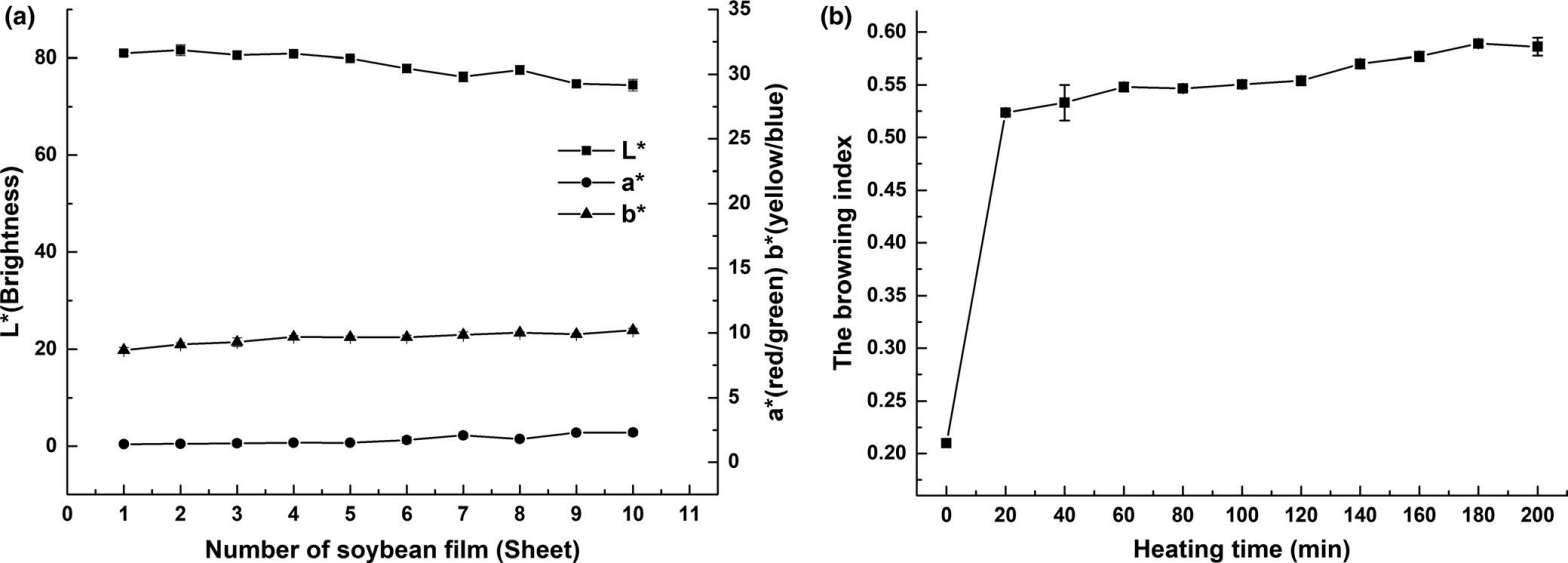
Color change in soymilk and soybean film during continuous peeling. (a) The varying of the chromatic aberration of soybean film. (b) Changes in browning index of soymilk

During the constant temperature (85°C) peeling process, the Maillard reaction occurred between the reducing sugars and the free amino groups of the protein in the soybean milk. It is observed from Figure 2a that the browning index of the soybean milk gradually increased as the holding time increased, which indicated that the black‐like substance in the soybean milk gradually accumulated during the peeling process. It showed that the Maillard reaction was constantly occurring as the holding time increased.

The color change in the soybean film during the peeling process was shown in Figure 2b, where L* value represented the brightness, a* value represented the red/green color, and the b* value represented the yellow/blue color. From the figure, we could find that L* value showed a downward trend, while a* value and b* value both decreased, and b* value decreased slightly compared with a* value, which indicated that the soybean film was successively revealed. During the continuous peeling, the brightness gradually decreased, and the proportion of yellow and red gradually increased, and it might be caused by the Maillard reaction occurred at the high temperature.

### 3.4 The changes in the Maillard reaction intermediate products

**FIGURE 3:**
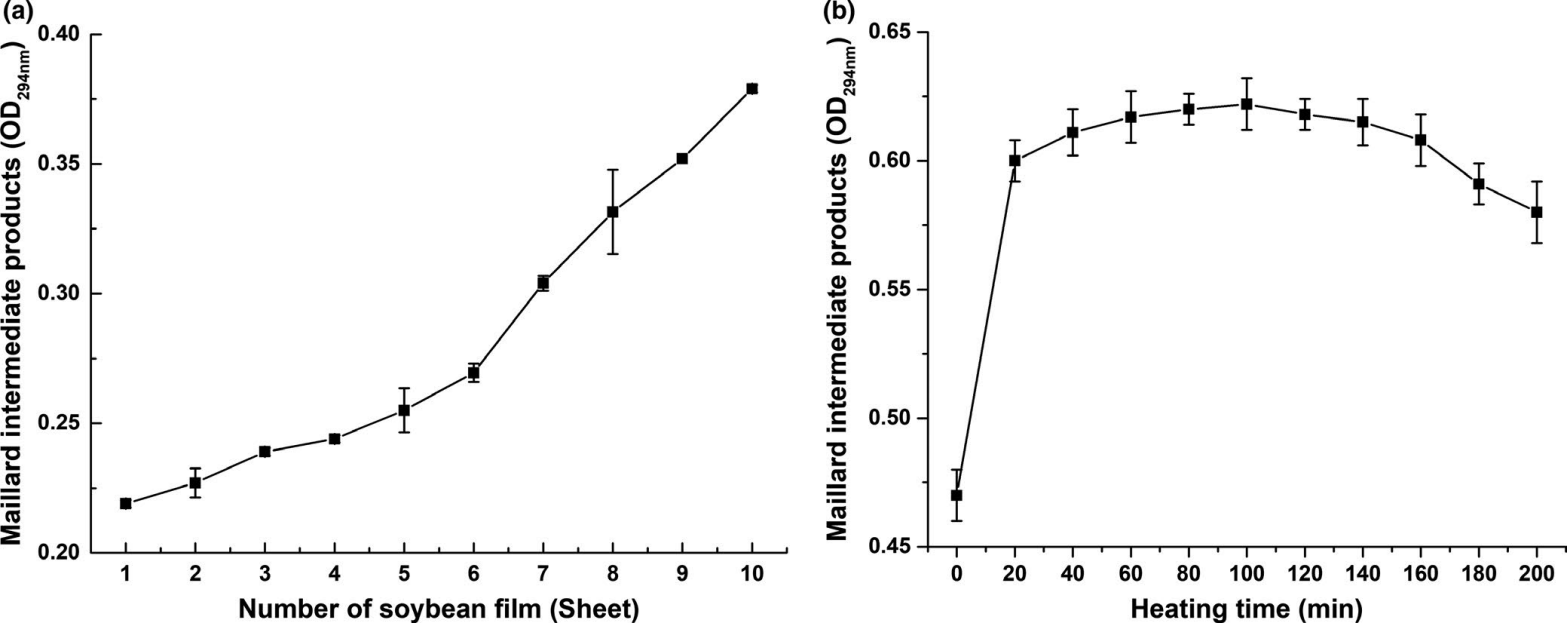
Changes in the Maillard intermediate products of (a) soybean film and (b) soymilk during the film peeling

In order to explain the color change in soybean film during peeling process, the Maillard reaction products were further analyzed. It can be seen in Figure 3a that the Maillard intermediate products could be detected in the soybean milk without heat treatment, and its content was significantly lower than that of soybean milk after heat treatment. This might be due to the Maillard reaction occurred during the pulping process in the beater. The Maillard reaction intermediates in soybean milk first increased and then decreased with the increase in the heat preservation time and reached the highest point at the 100 min (the fifth soybean film). At the beginning of heating process, Amadori rearrangement and Strecker degradation reaction generated some intermediate substances, such as aldehydes, ketones, and furans (Varoujan, 2003). After 100 min, these intermediate substances would undergo aldehyde‐amine condensation, hydroxyaldehyde condensation and other reactions to produce more advanced Maillard reaction products, such as unsaturated aldehyde‐ketones, and might be further converted into some flavorings. On the other hand, the aldehyde group‐amination reaction between these intermediate substances and amino compounds generated melanoidin substances. In Figure 2, the increasing of browning index represented the accumulation of melanoidin substances. Moreover, the accumulation rate was faster in the later period, indicating that the conversion rate of the Maillard reaction intermediates into melanoidin substances was accelerated in the later period, which also led to the rapid decline in the Maillard reaction intermediates in the later period.

Figure 3b shows the change in the Maillard intermediate products in the soybean film. It could be seen that the Maillard intermediate products in the soybean film increased during the peeling process. It indicated the accumulation process of the Maillard reaction products in the peeling process. The degree of the Maillard reaction was affected by many factors, including temperature, pH, and water content (Martinez‐Alvarenga et al., 2014). When the soybean film was formed on the surface of soymilk, the film and soymilk should be considered as two systems, a solid film system and a liquid soymilk system. In this way, when the water on the surface of soybean milk evaporated to form the film structure, the Maillard reaction products were brought into the soybean film from the soybean milk. When the soybean film had a complete membrane shape, the intermediate products of the Maillard reaction were formed by the reaction between proteins and the carbohydrates inside the membrane. The Maillard reaction was inactive at low water activity, and the coloring reaction was not obvious below 80°C as the molecular motion of amino and carboxyl compounds were inadequate. The surface temperature was lower than the internal temperature of soybean milk, which inhibited the browning reaction of the Maillard reaction; that is, the forming of intermediate products was faster than the forming of melanoidin substances. Figure 3 shows the difference in the accumulation and decomposition of intermediates in the Maillard reaction between the soybean milk and the film.

**FIGURE 4:**
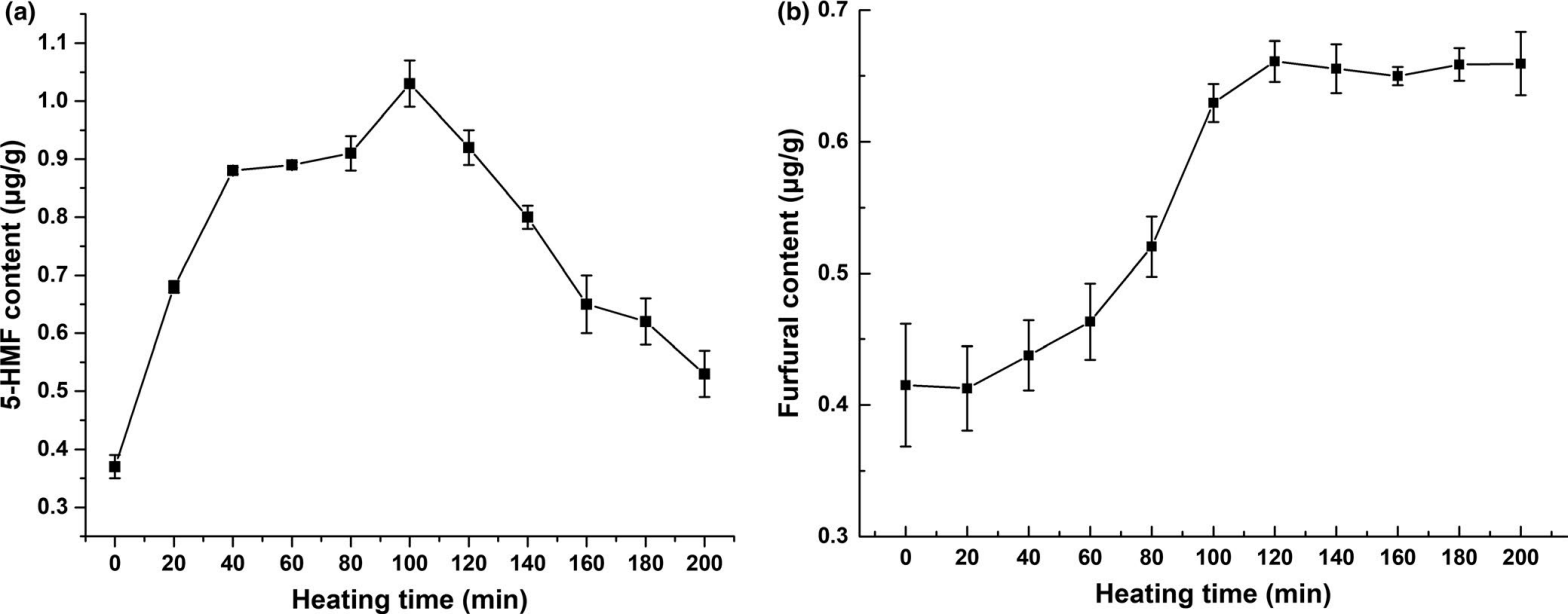
Changes in (a) 5‐HMF and (b) furfural of soymilk during the film peeling

It could be seen in Figure 4a that the content of 5‐HMF increased first and then decreased during the peeling time and reached a peak point at 100 min (the fifth film). It indicated that 5‐HMF was continuously generated and accumulated in the Maillard reaction before 100 min and then reacted with other substances to produce the Maillard end‐products such as melanin, and browning reaction occurred.

Figure 4b shows that the furfural content tended to rise in the early period and reached a peak value at 100–120 min. After that, there was no significant change. The change in furfural content indicated that after the accumulation of furfural in the early period, there was no significant difference between the accumulation rate and the consumption rate in the advanced period of the Maillard reaction, which led to the nonobvious change in furfural content in the later period.

The above results indicated that 5‐HMF and furfural produced by the Maillard reaction were continuously accumulated during the process of constant temperature peeling, and 5‐HMF began to be consumed after 100 min, while furfural tended to be stable. The accumulation of the Maillard reaction products also explained the change in soybean film color.

### 3.5 Analysis of mechanical properties of soybean film

**FIGURE 5:**
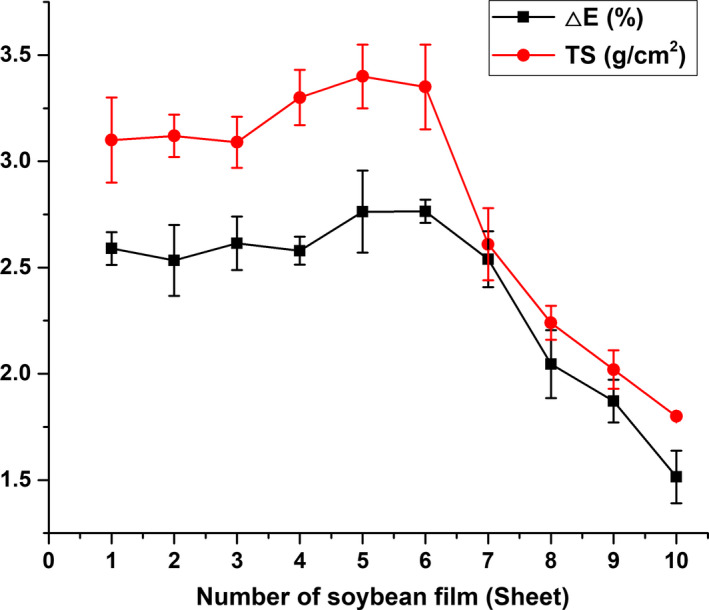
Changes in (a) TS and (b) Δ*E* of soybean film

Figure 5 shows the change in TS and ΔE of the film during the peeling process. It could be seen that there was no significant change in the TS and Δ*E* of the film formed in the early period, the TS was maintained at about 3.2 g/cm^2^, and the ΔE was maintained at about 2.7%. In the late period of film formation, the TS and Δ*E* showed significant downward trend, indicating that the mechanical properties of the later film deteriorated, which closely related to the composition of the film and soybean milk.

The soybean film is mainly composed of protein and lipid (Gallego et al., 2010). The network cross‐linking structure of the protein maintains the formation of the soybean film (Chang‐Yue, Hong‐Fang, Miao, & Xin‐Huai, 2016). In theory, when the protein content in the soybean film decreases, the network cross‐linking structure will become loose and resistant, and the strength of the film will become low (Petersson & Stading, 2005). In this study, in the early period of peeling, the protein content in the soybean milk was sufficient to form the film structure. Therefore, the influence of the membrane protein formed in the early period on the mechanical properties overshadowed the role played by other components. It led to a phenomenon in which the mechanical properties of the precorrosion film did not change significantly. In the late film formation process, the loss of protein in the soybean milk led to a decrease in the protein used to form the soybean film, and the protein cross‐linking structure increased due to the increase in the proportion of the components in the film formed by the carbohydrate and the fat. These factors led to a decrease in TS and Δ*E* of the films formed in the late period. Therefore, the mechanical properties of the soybean film were caused by changes in the microstructure due to changes in microscopic composition and mutual influence.

### 3.6 The change in dry matter lost and rehydration rate

**FIGURE 6:**
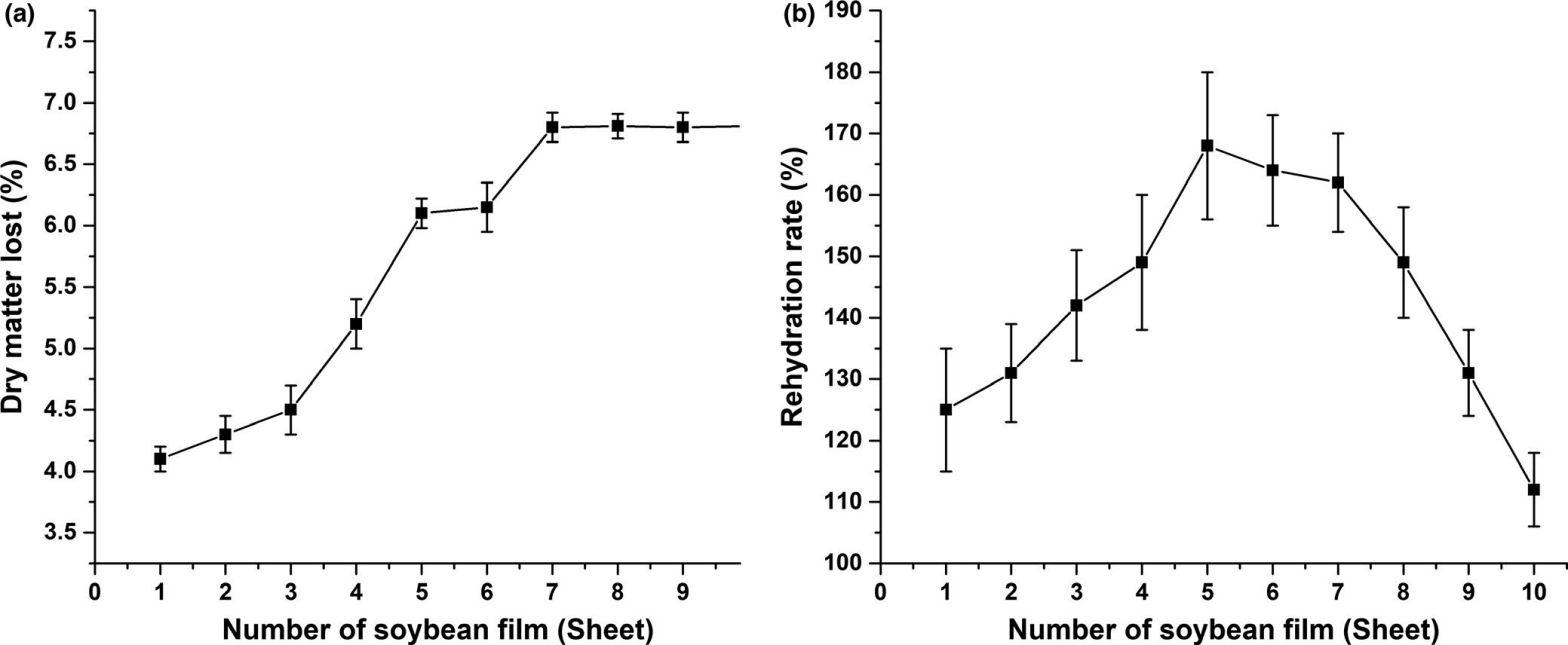
Changes in the rate of (a) dry matter lost and (b) rehydration of soybean film

Figure 6a shows the change in dry material loss rate of soybean film during peeling process, which showed a gradual upward trend and tended to be stable at the fifth film. The precious studies demonstrated that the protein content could affect the tightness of membrane structure, thus affecting the rate of dry matter loss (Gawel & Grzelak, 2020). In this study, the increase in dry matter lost might be due to the loose membrane structure caused by the decrease in protein content.

Figure 6b shows the change in rehydration rate of soybean film during peeling process. The rehydration rate presented a trend of first increasing and then decreasing, reaching the maximum value at the fifth film. The network of soybean film contained protein, lipid, and carbohydrate. The denaturation of protein affected the ability of the water absorption. The degree of protein denaturation was deepened under prolonged high temperature treatment in the late stage, which might be the reason that the rehydration rate of the soybean film became worse in the late period. Although carbohydrate had good water absorption, the content of carbohydrate in soybean film was relatively low, so carbohydrate had little influence on the rehydration rate of soybean film. In addition, when the protein network structure was relatively tight, the lipid was stable in the network structure. The decrease in protein content led to the looseness of membrane structure and the dissociation of lipid, which led to the decrease in rehydration rate of soybean film.

Combined with the above data, it could be seen that with the peeling process continued, the protein content decreased, leading the structure of the rotting skin to become loose, which caused the decrease in dry matter loss rate and water absorption of the soybean film.

### 3.7 Sensory evaluation results of soybean film

Table 4 shows the sensory evaluation results of soybean film. It could be seen that the color score of soybean film gradually decreased during the peeling process; that is, the color of the film became darker and lusterless, which was due to the Maillard reaction. In the terms of odor, there were abundant nutrients in soybean milk at the initial stage of peeling, and the reaction at the initial stage of the Maillard reaction could form a pleasant smell, so the soybean film had a nature bean flavor. The Maillard reaction produced an unpleasant flavor with prolonged heat retention, and loss of water and nutrient, resulting in a lower score. The taste and acceptability showed a trend of first increasing and then decreasing, and the taste of the fifth film was the best. The film had good toughness and was chewy. In general, the sensory evaluation results showed that the sensory performance of the continuous peeling film increased first and the decreased, and the fifth film had the best sensory quality.

**TABLE 4:** Sensory evaluation results of soybean film

	Color	Odor	Taste	Acceptability	Weighted score
B1	7.17 ± 0.29	6.00 ± 2.00	5.33 ± 1.53	5.33 ± 0.58	5.96 ± 0.86
B2	6.93 ± 0.12	6.00 ± 2.00	5.90 ± 1.15	5.50 ± 0.50	6.08 ± 0.61
B3	6.83 ± 0.29	7.00 ± 1.00	6.33 ± 1.04	5.33 ± 0.58	6.38 ± 0.75
B4	6.50 ± 0.50	6.93 ± 1.37	6.33 ± 1.53	6.00 ± 1.00	6.44 ± 0.39
B5	6.50 ± 0.50	7.00 ± 1.32	6.67 ± 1.53	6.17 ± 1.04	6.58 ± 0.35
B6	5.50 ± 0.50	6.33 ± 1.53	6.33 ± 1.15	6.33 ± 0.76	6.13 ± 0.42
B7	5.13 ± 0.23	6.17 ± 1.26	5.67 ± 1.53	5.00 ± 1.00	5.49 ± 0.53
B8	5.00 ± 0.31	6.00 ± 1.73	5.17 ± 1.26	5.00 ± 1.00	5.29 ± 0.48
B9	3.93 ± 0.90	5.83 ± 1.44	5.83 ± 0.76	4.50 ± 1.32	5.03 ± 0.96
B10	3.83 ± 0.76	4.83 ± 2.36	5.33 ± 0.58	4.33 ± 1.15	4.58 ± 0.65

### 3.8 Correlation analysis between the content of main nutrient and sensory evaluation index

As shown in Table 5, the protein and the four indicators had significant positive correlation relationship (correlation coefficient “*r* value” was .809**, .816**, .670*, and .760*, respectively). As one of the raw materials of the Maillard reaction, protein affected the color and the odor of soybean film, and the looseness of protein network structure affected the taste. The results showed that the test subjects were more receptive to samples with high protein and lipid content.

**TABLE 5:** Correlation between main nutritional components and sensory evaluation indexes of soybean film

	Color	Odor	Taste	Acceptability
Protein	0.809**	0.816**	0.671*	0.760*
Lipid	0.985**	0.662*	0.484	0.691*
Carbohydrate	−0.591	0.049	0.173	0.106

** was significantly correlated at the level of .01, * was a significant correlation at the level of .05.

The correlation between lipid and soybean film might be due to the attachment of lipid particles to the surface of the film, which affected the color. The smell of lipid would affect the flavor of the soybean film. There was a significant positive correlation between lipid content and the acceptability (the *r* value was .691*). There was no significant relationship between carbohydrate and the four sensory indexes.

### 3.9 Correlation analysis of color difference with the sensory evaluation index and the nutrient composition

It could be seen from Table 6 that the brightness (L* value) of color difference had a significant positive correlation with lipid and protein content (the *r* value was .798** and .969**, respectively). The redness (a* value) had a significant negative correlation with lipid and protein content (the *r* value was −.779** and −.954**, respectively). The yellowness (b* value) had a significant correlation with carbohydrate (*r* value was .824**). The color difference and the three indexes of color evaluation in the sensory indicators were significantly correlated (*r* value was .978**, −.969**, and −.841**, respectively), indicating that the sensory evaluation of the soybean film was correlated with the color difference measured by the color difference instrument. The color difference value (L* value) was significantly positively correlated with the acceptability, while a* was significantly negatively correlated with the acceptability.

**TABLE 6:** Correlation between color difference, sensory evaluation index, and nutritional components of soybean film

	Nutritional components			Sensory evaluation index			
	Protein	Lipid	Carbohydrate	Color	Odor	Taste	Acceptability
Color difference							
L*	0.798**	0.969**	−0.581	0.978**	0.657*	0.410	0.671*
a*	−0.779**	−0.954**	0.510	−0.969**	−0.677*	−0.416	−0.732*
b*	−0.521	−0.861**	0.824**	−0.841**	−0.315	−0.100	−0.349

** was significantly correlated at the level of .01, * was a significant correlation at the level of .05.

### 3.10 Soybean film quality evaluation system

The sensory indexes, mechanical property (TS and Δ*E*), dry matter lost, rehydration rate, and nutrient components of soybean film were incorporated into the evaluation model, and principal component analysis was performed on these data. Two main components of the quality evaluation were obtained (Table 7).

**TABLE 7:** Correlation between mechanical properties, dry matter lost, rehydration rate, sensory evaluation index, and nutritional components of soybean film

	Nutritional components			Sensory evaluation index			
	Protein	Lipid	Carbohydrate	Color	Odor	Taste	Acceptability
Dry matter lost	−0.558	−0.905**	0.873**	−0.884*	−0.324	−0.156	0.374
Rehydration rate	0.673*	0.279	0.471	0.299	0.760*	0.614	0.683*
Δ*E*	0.893**	0.841**	−0.126	0.814**	0.814**	0.645*	0.864**
TS	0.867**	0.869**	−0.167	0.847**	0.793**	0.711*	0.934*

** was significantly correlated at the level of .01, * was a significant correlation at the level of .05.

After principal component analysis, it could be seen that among the two principal components, the principal component 1 accounted for 48.436% of the original information and the principal component 2 accounted for 42.120%, and the cumulative variance contribution rate was 90.556%, indicating that these two principal components could replace the above several quality characteristics to evaluate the quality of soybean film (Table 8).

**TABLE 8:** Eigenvalue and variance contribution rates

Principal component	Eigenvalues	Variance contribution (%)	Accumulative variance contribution (%)
1	7.265	48.436	48.436
2	6.318	42.120	90.556

By calculating the factor load matrix in Table 9 and the original data after standardization, the comprehensive score of each rotting skin can be obtained. Because the weight of each principal component was different, it was taken as a factor according to the weight ratio. That is, the calculation formula could be expressed as the comprehensive score (Equation (5)).

(5) $$F=0.5348f_{1}+0.4651f_{2}$$

**TABLE 9:** Component matrix

Index	Principal component	
	1	2
Protein	−0.490	0.787
Lipid	−0.863	0.496
Carbohydrate	0.902	0.351
Rehydration rate	0.161	0.907
Dry matter lost	0.989	−0.095
Δ*E*	−0.483	0.834
TS	−0.531	0.819
L*	−0.841	0.473
a*	0.806	−0.521
b*	0.953	−0.103
Color	−0.860	0.489
Odor	−0.258	0.882
Taste	−0.032	0.839
Acceptability	−0.284	0.880

Composite scores of soybean film are shown in Table 10. The 10 films were ranked according to the score. The results showed that the comprehensive quality of the soybean film obtained in the early stage of peeling was worse than that in the middle stage. In general, the score indicated that the soybean films obtained in the middle stage (the 5th, 6th, and 7th pieces) had better comprehensive quality.

**TABLE 10:** Comprehensive score of the main factors of soybean film

Soybean film	Score	Rank
B1	−5.125	10
B2	−3.212	9
B3	−1.514	8
B4	1.689	4
B5	2.428	1
B6	2.337	2
B7	2.092	3
B8	0.997	5
B9	0.447	6
B10	−0.041	7

The comprehensive scores of the two principal components obtained above were used as variables to conduct systematic cluster analysis on the 10 films, and the results are shown in Table 11. As shown in Tables 10 and 11, the fourth, fifth, and sixth films were divided into one category, they were classified as first‐class film. Similarly, the seventh, the eighth, and the ninth films were classified as second‐class film. The first, the second, and the third films were classified into one category, and the tenth film was classified into a separate category.

**TABLE 11:** Results of cluster analysis

Classification	Number of soybean film
1	4, 5, 6
2	7, 8, 9
3	1, 2, 3
4	10

### 3.11 Characterization of soybean film microstructure by SEM

**FIGURE 7:**
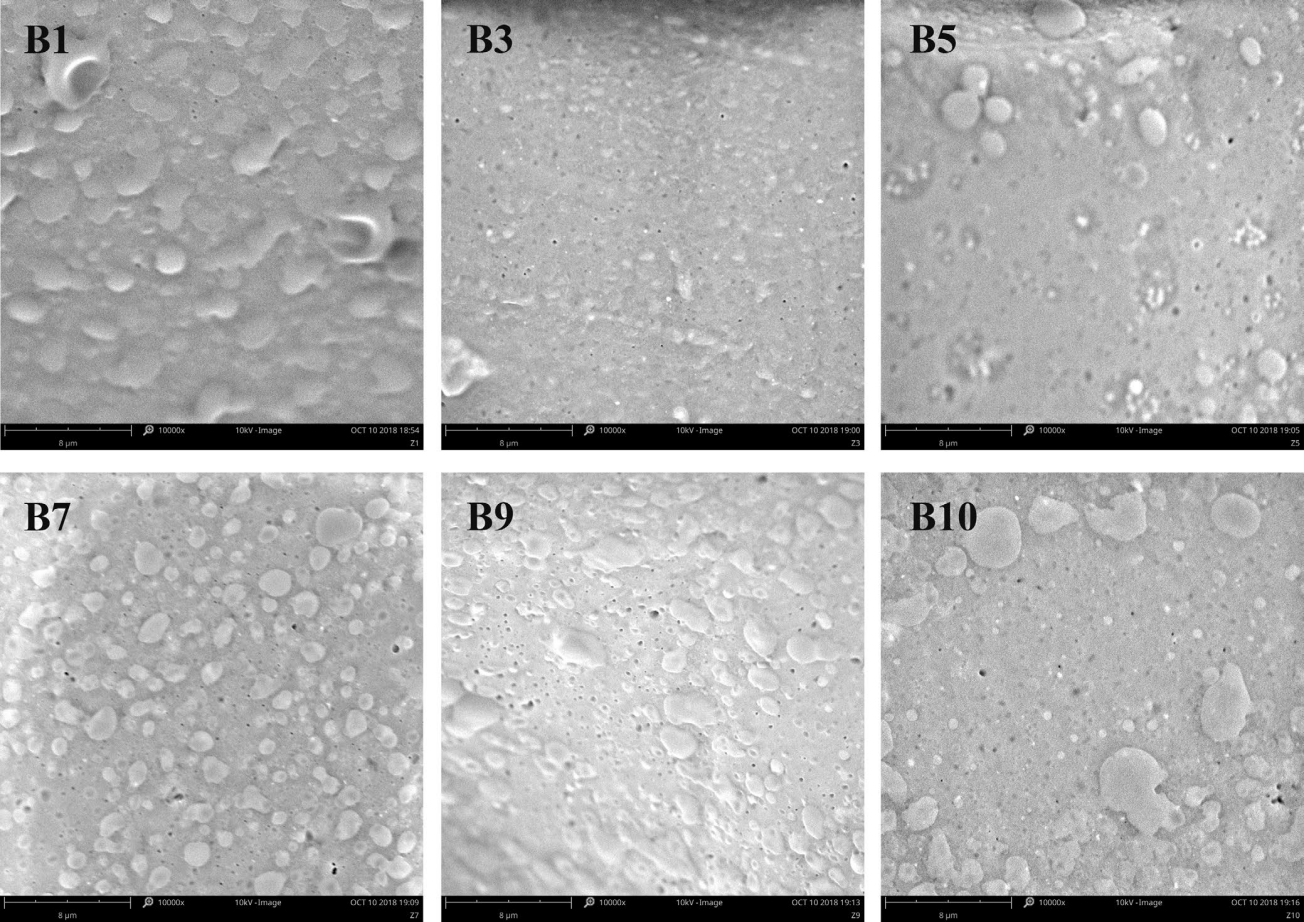
Microstructure of soymilk during the film picking (10,000×)

The first, third, fifth, seventh, ninth, and tenth soybean films were selected for microstructure analysis by electron microscopy. It could be seen from Figure 7 that the surface of the soybean film was not smooth, and there were many white particles of different sizes; especially in the film formed in the late period, very small white globules (<100 nm) had been observed. It was considered to be protein particles or some soluble protein (Yeming & Tomotada, 2010). In addition to white particles, black‐gray pores (<500 nm) could be seen, which might be lipid particles in the soybean film. Micropores formed on the surface of the film due to evaporation of water. As the peeling process continued, these black‐gray holes increased, further leading to a decrease in the flatness of the film. In the later period of film formation, the film became less permeable and the microstructure became more loose, resulting in the failure of film formation.

## 4 CONCLUSIONS

In this paper, the changes in the composition of soymilk in the process of traditional soybean film processing were studied. In the process of multiple peeling, the surface moisture of soybean milk evaporated, protein and fat were the main components of soybean film. A film formed on the surface of the soybean milk with a network structure formed by protein denaturation. During the formation of soybean film, protein was the most expended component, lipid was consumed relatively less, and carbohydrate was consumed the least.

At the initial period, the nutrients in the soymilk were sufficient, the protein was denatured, the sulfhydryl group was exposed, and the network structure was easy to form. It led to the formation of the soybean film, the structure was relatively tight. However, with the decrease in the content of 7S protein, 11S protein, and lipid in soybean milk, and the prolonged high temperature heating, protein denatured and polymerized to form larger molecules. The viscosity of soybean increased, and the ability of protein to form network structure deceased. In the early period, the proportion of carbohydrates increased, and it had the function of softening the network structure, and the small molecules of the Maillard reaction intermediate products accumulated and were gradually consumed to form polymer compounds, leading to the deepening of the color of the soybean film and a decline in the quality index of the film formed in the later period.

The four sensory indexes (color, smell, taste, and acceptability) of the soybean film were measured, and the weighted score of each index was the highest in the fifth film. The sensory evaluation results showed that the sensory performance of the skin was first increased and then decreased after repeated peeling, and the sensory quality of the fifth film was the best. Through correlation analysis, this study found that protein and lipid contents in soybean film were significantly related to the acceptance, which might be related to the change in color, odor, and taste caused by the Maillard reaction. Through further clustering analysis, this study found that the soybean film obtained in the middle period of peeling had the best quality.

This research provided a deeper understanding of the changes in the quality of soymilk and soybean film during the processing of traditional soybean film, providing a good theoretical basis to industrialized large‐scale production of soybean film.

## CONFLICT OF INTEREST

The authors declare there are no conflicts of interest regarding the publication of this paper.

## AUTHOR CONTRIBUTIONS

Yin‐Yi Ding drafted the manuscript. Juanjuan Li collected some test data. Cheng Wu collected test data. Zhengyu Gu developed an experimental plan. Yuexi Yang participated in the designation of the experimental protocol.
